# Quantitative effects of tobacco smoking exposure on the maternal-fetal circulation

**DOI:** 10.1186/1471-2393-11-24

**Published:** 2011-03-31

**Authors:** Julia de B Machado, Plínio VM Filho, Guilherme O Petersen, José M Chatkin

**Affiliations:** 1 Obstetrics, Sao Lucas Hospital da Pontificia Universidade Catolica do Rio Grande do Sul,of Rio Grande do Sul, Porto Alegre, Brazil; 2 Pharmacy student of the Pontificia Universidade Catolica do Rio Grande do Sul, Porto Alegre, Brazil; 3 Pulmonary Disease, Sao Lucas Hospital, School of Medicine, Pontificia Universidade Catolica do Rio Grande do Sul , Brazil

## Abstract

**Background:**

Despite the existence of various published studies regarding the effects of tobacco smoking on pregnancy, and especially in regards to placental blood flow and vascular resistance, some points still require clarification. In addition, the amount of damage due to tobacco smoking exposure that occurs has not been quantified by objective means. In this study, we looked for a possible association between flow resistance indices of several arteries and the levels of urinary cotinine and the concentration of carbon monoxide in the exhaled air (COex) of both smoking and non-smoking pregnant women. We also looked for a relationship between those findings and fetal growth and birth weight.

**Methods:**

In a prospective design, thirty pregnant smokers and thirty-four pregnant non-smokers were studied. The volunteers signed consent forms, completed a self-applied questionnaire and were subjected to Doppler velocimetry. Tobacco smoking exposure was quantified by subject provided information and confirmed by the measurement of urinary cotinine levels and by the concentration of carbon monoxide in the exhaled air (COex). The weight of newborns was evaluated immediately after birth.

**Results:**

Comparing smoking to non-smoking pregnant women, a significant increase in the resistance index was observed in the uterine arteries (P = 0.001) and umbilical artery (P = 0.001), and a decrease in the middle cerebral artery (P = 0.450). These findings were associated with progressively higher concentrations of COex and urinary cotinine. A decrease in the birth weight was also detected (P < 0.001) in association with a progressive increase in the tobacco exposure of the pregnant woman.

**Conclusions:**

In pregnant women who smoke, higher arterial resistance indices and lower birth weights were observed, and these findings were associated with increasing levels of tobacco smoking exposure. The values were significantly different when compared to those found in non-smoking pregnant women. This study contributes to the findings that smoking damage during pregnancy is dose-dependent, as demonstrated by the objective methods for measuring tobacco smoking exposure.

## Background

Evidence regarding the negative effects of tobacco smoking on fetal development is widely documented in existing literature. The toxic effects vary from perinatal complications, such as low birth weight, to changes in adult behavior [1-5].

Regarding the effects of maternal tobacco smoking on placental blood flow and vascular resistance, there still exists some controversy regarding which vessels are most affected and if the effects are acute or chronic [6].

However, most studies quantify smoking only by the self-report of subjects, a method that is frequently not reliable. It is known that smokers sometimes provide false information about their smoking status, and among pregnant women, the percentage of misreporting is unknown. This occurs especially if the individual is under pressure to quit smoking and thus a bias towards a socially desirable response may occur [7-9].

The prevalence of patients who state they are not smoking anymore when, in fact, they actually still do varies between 3% and 10% [9,10]. Abstinence during smoking cessation treatment needs to be confirmed by objective measurements and biochemical markers have been introduced in clinical practice [9,10].

The carbon monoxide concentration in exhaled air (COex) is the most frequently used marker [10]. It is affordable and measured by a non invasive technique that provides immediate and reliable results [11]. It has been widely used at smoking cessation centers in most countries including Brazil [12-14].

The salivary, plasmatic and urinary cotinine concentrations are considered to be the golden standard for objective evidence, but measurements require expertise [9], are expensive and are not for clinical practice [15].

Doppler velocimetry in Obstetrics is used to measure blood flow in the uterine arteries, and it characterizes the blood flow from the mother to the fetus. When the blood flow is studied in the fetal middle cerebral artery, it shows the flow in fetal brain. There are tables with established values that are considered normal. The methodology has already been standardized and we have followed such guidelines in this study.

This study aimed to search for a relationship among resistance indices of the uterine, umbilical and middle cerebral fetal arteries via Doppler velocimetrical ultrasonographic evaluation and smoking habits measured through objective methods (cotinine concentration and COex). The relationship between smoking and fetal growth and with birth weight was also examined.

Our primary objective was to assess whether there is a proportional change in vascular resistance as quantitative exposure to smoking during pregnancy increases. The hypothesis is that smoking reduces the maternal-fetal blood flow related to increased tobacco smoking and effects fetal growth.

## Methods

Using a prospective cohort design study, 30 pregnant smokers and 34 pregnant non-smokers were recruited between September 2008 and September 2009 from the Low Risk Prenatal Care Center of the Obstetric Department at the Hospital Sao Lucas da Pontificia Universidade Catolica do Rio Grande do Sul (HSL-PUCRS) in Porto Alegre, Brazil.

The inclusion criteria were: pregnant women between 18 and 35 years of age, in the third trimester of gestation (dated in the first trimester), regularly receiving prenatal care, had no gestational complaints and agreed to participate in the study by signing the consent form. The exclusion criteria were: ultrasonographic diagnosis of fetal malformations, clinical situations related to the pregnancy or any other disease that could interfere with fetal growth, illiteracy, psychiatric disorders, alcoholism, former smokers and the possibility to move to another city and to lose contact during the study. Patients who discontinued prenatal care and those that stopped or started smoking during their pregnancy were also excluded. No patient was excluded after inclusion in the study because no patient discontinued the follow up during the prenatal period.

Smoking was determined according to the report of using cigarettes from the periconceptional period to the day of the interview. The periconceptional period was defined as the period starting one month before the date of the last menstruation. To be considered a non-smoker, patients must have stated that they had never smoked, especially during their pregnancy, and reports were confirmed by a biochemical marker.

All patients were submitted to same tests to confirm their smoking status: concentration of carbon monoxide in the exhaled air and concentration of urinary cotinine. We found no discrepancy, that is, no patient who said was a non-smoker was detected as a smoker.

At the initial medical visit, the objectives of the study were explained and the consent form signature was obtained.

Next, a standardized questionnaire was completed, pregnancy and smoking data were collected and the COex measurement was performed. A urine sample was also collected to measure cotinine levels, and an ultrasonograph examination was performed. Smoking between the tests was not permitted. After the birth, patient was contacted to collect data about the newborn one month after delivery.

Doppler velocimetry exam with colored mapping of the uterine arteries, umbilical arteries and fetal medium cerebral arteries was performed with an Ultrasonix machine (Sonix Model, Version 2.5, Ultrasonix Medical Corporation, Canada), and the resistance indices (RI) were recorded. We also recorded the amniotic fluid index, the placental grade, fetal biometry and the estimated fetal weight placed on a percentile curve for the gestational age to adjust for possible bias.

To decrease possible bias at ultrasonography due to a large variability of Doppler values, three measurements were taken, and the mean was recorded.

COex was measured by a MicroCO Meter (MicroCO - Micro Medical Ltd., Rochester, Kent, UK) using an electrochemical sensor. Patients were instructed to breathe deeply, hold their breath for 20 seconds and then to exhale slowly and completely through a mouthpiece. Smokers had COex measured no more than two hours after their last smoked cigarette [9].

For measurement of the cotinine concentration in the urine, a previously-validated high performance liquid chromatography (HPLC) method was used [16].

Whereas the ultrasonography examinations and the exhaled CO measurements were performed by the same author (JBDM), the cotinine measurements were performed at the PUCRS Toxicology Institute by a technician blind to patient study group.

Mean ± SD were used to describe the symmetric, continuous data and the median and range was used for asymmetric data. A percentile calculation was used for categorical data. Student's t test and ANOVA were used to compare means. Adjustments for potential confounding effects were made by covariance analysis (ANCOVA) and multiple linear regression. Possible confounding factors included in the analysis for adjustment were patient age, gestational age, degree of placenta, amniotic fluid index, number of pregnancies. The assigned level of significance was 0.05. The data were processed by the Statistical Package for the Social Sciences (SPSS) Version 17.0

Patients were divided into four groups according to the levels of COex (Group1: 0 ppm; Group 2: 1-4 ppm; Group 3: 5-9 ppm; Group 4: ≥10 ppm) in order to analyze possible changes in the resistance indices. The same analyses were also performed according to the levels of urinary cotinine (Group 1: 0 ng/mL; Group 2: <50 ng/mL; Group 3: 50-500 ng/mL; Group 4 >500 ng/mL).

We used percentiles to compare the resistance indices and fetal birth weights according to the gestational age of each patient. For weight, a Hadlock table was used [17]. Analysis of the resistance index values was performed by using the published standards for the respective percentiles [17].

The study was approved by the PUCRS Research Ethics Committee, under No. 08/04319 on May 09^th ^2008.

## Results

Thirty pregnant smokers and 34 pregnant non-smokers women were included in this study analysis. No significant difference was observed between the two groups in regards to age, amniotic fluid index, cultural and social status, number of prior miscarriages or placenta grade. Smokers had a greater number of pregnancies and births than non-smokers, with values of 3.0 [95%CI 3.0-11.0] vs. 2.0 [95%CI 1.0-5.0] p = 0,04; 1.5 [95% CI 0.0-7.0] vs. 1.0 [0.0 - 4.0] p = 0,04 respectively (Table 1).

**Table 1:** Characteristics of the sample according to smoking habits

	Non smokers n = 34	Smokers n = 30	P
Characteristics			
Age	27.6 ± 6.1	27.3 ± 5.2	0.82
Pregnancies	2.0 [1 a 5]	3.0 [3 a 11]	0.04
Parities	1.0 [0 a 4]	1.5 [0 a 7]	0.04
Abortions	0.0 [0 a 3]	0.0 [ 0 a 3]	0.62
AFI	14.0 ± 2.0	13.3 ± 2.8	0.21
Placenta grade	1 (56%) [0 a 2]	1(70%) [0 a 3]	0.31

Data are presented as mean ± SD or median [minimum and maximum]. AFI: amniotic fluid index. Age (years), pregnancies, abortions and parities (number), Amniotic Fluid Index(cm), Placenta grade percent type I (As classification Granumm)^17^. P: statistical significance

When the entire group of non smokers was studied, the RI in both uterine arteries (right uterine 37.3 ± 20.0 vs. 58.0 ± 23.0 p = 0.0010; left uterine 38.9 ± 17.6 vs. 56.1 ± 19.6 p = 0.001) and the umbilical artery (43.0 ± 19.0 vs.65.0 ± 15.0 p = 0.001) were higher compared to smokers. The RI in the middle cerebral artery was lower (45.00 ± 15.00 vs.36.3 ± 21.0 p = 0.450), but, the difference was not statistically significant (Table 2).

**Table 2:** Percentile of RI among pregnant women according to smoking habits

	Non smokers n = 34	Smokers n = 30	P
RI Percentile			
RUA	37.3 ± 20.0	58.0 ± 23.0	0.001
LUA	38.9 ± 17.6	56.1 ± 19.6	0.001
UA	43.0 ± 19.0	65.0 ± 15.0	0.001
MCA	45.0 ± 15.0	36.3 ± 21.0	0.450

Data are presented as mean ± SD. P: statistical significance.

RUA: right uterine artery, LUA: left uterine artery,UA: umbilical artery, MCA: middle cerebral artery. RI: vascular resistance index.

When the participants were grouped according to COex levels (Group1 to Group 4), progressively higher RI values were observed for the right uterine (Group 1: 36.2 ± 21.8, Group 2: 46.9 ± 20.3, Group 3: 44.4 ± 28.9, Group 4: 66.5 ± 17.5 , p < 0.001) and left uterine arteries (Group 1: 39.5 ± 18.9, Group 2: 40.5 ± 16.8, Group 3: 52.8 ± 19.5, Group 4: 66.1 ± 16.7, p < 0.001) and the umbilical artery (Group 1: 44.5 ± 21.3, Group 2: 49.1 ± 17.9, Group 3: 61.1 ± 13.1, Group 4: 71.1 ± 16.2, p < 0.001) as the mean COex increased. In addition, we observed lower RI values of the middle cerebral artery (Group 1: 46.4 ± 14.3, Group 2: 42.1 ± 17.1, Group 3: 47.2 ± 19.5, Group 4: 27.7 ± 22.2, p < 0.001) as COex increased (Table 3).

**Table 3:** Percentile of RI and of birth weight between four groups according to carbon monoxide exhaled concentration

	COex 0 ppm n = 21	COex 1 - 4 ppm n = 21	COex 5 - 9 ppm n = 9	COex ≥ 10 ppm n = 13	***P***^**[1]**^	***P***^**[2]**^
RI Percentile						
RUA	36.2 ± 21.8 [10 a 90]	46.9 ± 20.3 [10 a 75]	44.4 ± 28.9 [10 a 90]	66.5 ± 17.5 [25 a 90]	0.003	<0.001
LUA	39.3 ± 18.7 [25 a 75]	40.5 ± 16.8 [25 a 75]	52.8 ± 19.5 [25 a 75]	66.1 ± 16.7 [50 a 95]	<0.001	<0.001
UA	44.5 ± 21.3 [10 a 75]	49.1 ± 17.9 [5 a 75]	61.1 ± 13.1 [50 a 75]	71.1 ± 16.2 [50 a 95]	0.001	<0.001
MCA	46.4 ± 14.3 [25 a 75]	42.1 ± 17.1 [5 a 75]	47.2 ± 19.5 [25 a 75]	27.7 ± 22.2 [5 a 75]	0.022	<0.001
Percentile of birth weight	49.05 ± 22.7 [10 a 90]	49.90 ± 25.8 [3 a 90]	41.2 ± 25.3 [3 a 75]	25.1 ± 16.6 [3 a 50]	0.017	<0.001

Data are represented as mean ± SD [minimum, maximum]. COex: concentration of carbon monoxide in the exhaled air, RI: vascular resistance index, RUA: right uterine artery, LUA: left uterine artery, UA: umbilical artery, MCA: middle cerebral artery. P: statistical significance, [1]: variance analysis - ANOVA - *oneway*, [2]: linear tendency obtained in regression model.

When analyzing the increasing values of urinary cotinine in the four groups of smokers, a statistically significant progressive increase of the resistance indices for the left uterine artery (p = 0.037) and the umbilical artery (p < 0.001) were also identified. Although not statistically significant, the same trend was observed for the right uterine artery p = 0.267 (Table 4). There was, however, a decreasing trend for the RI of the middle cerebral artery related to an increasing concentration of urinary cotinine. This finding was statistically significant in the linear regression, but the significance was not maintained when adjusting for the possible confounding factors described above.

**Table 4:** Percentile of RI and of birth weight between four groups according to urinary cotinine levels

	Cotinine 0 ng/mL n = 30	Cotinine < 50 ng/mL n = 10	Cotinine 50 - 500 ng/mL n = 14	Cotinine >500 ng/mL n = 10	***P***^**[1]**^	***P***^**[2]**^
RI Percentile						
RUA	42.8 ± 22.8 [10 a 90]	40.0 ± 25.7 [10 a 90]	55.3 ± 23.5 [10 a 90]	22.9 ± 22.9 [25 a 75]	0.201	0.267
LUA	41.1 ± 20.8 [25 a 95]	42.5 ± 20.6 [25 a 75]	58.9 ± 15.8 [25 a 75]	52.5 ± 18.4 [25 a 75]	0.033	0.037
UA	46.8 ± 20.1 [5 a 75]	46.0 ± 21.0 [10 a 75]	63.5 ± 14.6 [50 a 90]	68.5 ± 17.3 [50 a 95]	0.003	0.001
MCA	45.3 ± 15.7 [5 a 75]	42.5 ± 16.8 [25 a 75]	36.8 ± 21.8 [5 a 75]	34.5 ± 23.8 [5 a 75]	0.321	0.14
Percentile of birth weight	45.6 ± 21.5 [3 a 90 ]	62.4 ± 24.4 [25 a 90]	36.6 ± 24.0 [3 a 90]	27.1 ± 22.8 [3 a 75]	0.006	0.007

Data are represented as mean ± SD [minimum, maximum]. RI: vascular resistance index, RUA: right uterine artery, LUA: left uterine artery, UA: umbilical artery, MCA: middle cerebral artery. P: statistical significance, [1]: variance analysis - ANOVA - *oneway*, [2]: adjusted for age, number of pregnancies and AFI in ANCOVA model.

There was a significant decrease (p < 0.001) in the percentile of the fetal birth weight in relation to increased mother's COex as well as increased urinary cotinine values ( p = 0.007).

## Discussion

This study showed that the resistance indices of the placental vascular bed tended to rise with a quantitative increase in tobacco smoking exposure measured by objective methods in a dose-dependent effect. As far as we know, this is the first report showing such dose-effect through objective measurements of tobacco smoking exposure.

Smoking women were classified according to progressively increasing levels of COex to look for an association of those concentrations with an increase in resistance indices. Indeed, an increase in COex levels was associated to an increase RI of the uterine and umbilical arteries and a decrease in the resistance of the middle cerebral artery. These findings were all statistically significant, even after adjusting for possible confounding factors.

When the groups were analyzed according to increasing values of urinary cotinine, another marker of tobacco exposure tested in this study, significant increases were also observed in the left uterine and umbilical arteries. The right uterine artery showed a trend towards an increased RI, however, not statistically significant. The middle cerebral artery showed a tendency towards a decreased resistance related to the increasing cotinine measurements, not statistically significant.

Smoking during pregnancy is known to influence or even determine low birth weight and it is the main preventable cause of restricted intrauterine growth and small size for gestational age [18]. Our findings are in accordance of previous reports, showing that as a consequence of the increase in resistance indices, the decrease amount of blood and oxygen transported to the fetus resulted in lower weights of the newborns.

This study contributes to those reports showing that it is probably a dose-dependent effect. We found that decreased birth weights correlated with increase in tobacco smoking exposure, confirmed by both exposure methods, urinary cotinine and COex.

A pattern of maternal-fetal perfusion with characteristics of chronic hypoxia among the pregnant smokers was observed. Those findings were similar to the centralization phenomenon [19], with an increase in the RI of the umbilical artery and a decrease in RI of the middle cerebral artery. Thus, we infer that the fetus of a smoking mother, as a consequence of the decreased placental blood flow and possible tissue hypoxia, compensates with tendency towards centralization of the blood flow.

Although the details of these circulatory adjustments and their mechanisms are incomplete, it is likely that when the partial pressure of O_2 _decreases and that of CO_2 _increases above a certain level, the aortic and carotid chemoreceptors are activated. This probably is the mechanism that regulates the central vasodilatory response to guarantee adequate oxygenation to the fetal brain [20].

Prior studies [6,19,21,22] already studied the repercussions of smoking on maternal-fetal flow, but all have used patients' self-report to quantify tobacco smoking exposure. As mentioned previously, data reported by smoking patients are not always reliable, what might justify the heterogeneity of the results found in those studies[23].

Albuquerque and colleagues [19] compared 74 pregnant smokers with 69 controls, classifying then according to smoking status as informed by the patients. They concluded that chronic maternal smoking was associated with the increasing resistance of the uterine, umbilical and fetal middle cerebral arteries. Other studies [20,21] evaluated the cigarette acute effects. Kimya et al. [6] studied 22 pregnant smokers and 21 non-smokers by measuring Pulsatility Index, Resistance Index and Systole/Diastole of the uterine and umbilical before and after smoking a cigarette default. They found no significant change in the Doppler that could be attributed to the acute effect of cigarette smoking, but all indices were significantly higher in the smoking group compared with the control group both before and after cigarette smoking. They conclude that chronic smoking caused an increase in vascular resistance of the placenta and umbilical cord.

One of the strengths of this study is that the smoking status of the patients were confirmed by biochemical quantitative measurements (concentration of exhaled CO and of cotinine in a urine sample), showing a possible dose-effect of tobacco smoking exposure on maternal-fetal blood perfusion.

Our results may be used as a tool for helping pregnant smokers to stop or at least to decrease their tobacco exposure by emphasizing that the risks to the fetus occur in a dose-dependent manner. Our objective data demonstrate that a higher smoking level results in greater hemodynamic effects on the fetus. These effects culminate in growth restriction that may have multiple effects throughout the life of children.

## Conclusions

In this study, using Doppler velocimetry in uterine, umbilical and middle cerebral arteries flow, changes in resistance indeces were detected comparing smoking to non smoking pregnant women. A dose-dependent effect in placental blood flow was found such that resistance indices increased in accordance with increasing levels of tobacco smoking exposure.

In addition, we found a decrease in fetal birth weight that also correlated with an increase in measurements of COex and urinary cotinine.

## Competing interests

The authors declare that they have no competing interests.

## Authors' contributions

JDBM: physician, principal investigator and responsible writing the first draft of the paper. PVM: physician, co-investigator, responsible for the patients. GOP: chemistry student, responsible for the biochemical techniques. JMC: physician, responsible for the statistical analyses and for writing and reviewing the manuscript. All authors read and approved the final manuscript.

## Pre-publication history

The pre-publication history for this paper can be accessed here:

http://www.biomedcentral.com/1471-2393/11/24/prepub
